# Incidence, Predictive Factors, and Clinical Outcomes of Acute Kidney Injury after Gastric Surgery for Gastric Cancer

**DOI:** 10.1371/journal.pone.0082289

**Published:** 2013-12-09

**Authors:** Chang Seong Kim, Chan Young Oak, Ha Yeon Kim, Yong Un Kang, Joon Seok Choi, Eun Hui Bae, Seong Kwon Ma, Sun-Seog Kweon, Soo Wan Kim

**Affiliations:** 1 Department of Internal Medicine, Chonnam National University Medical School, Gwangju, Korea; 2 Department of Preventive Medicine, Chonnam National University Medical School, Gwangju, Korea; 3 Jeonnam Regional Cancer Center, Chonnam National University Hwasun Hospital, Hwasun-gun, Republic of Korea; IRCSS - Istituto di Ricerche Farmacologiche Mario Negri, Italy

## Abstract

**Background:**

Postoperative acute kidney injury (AKI), a serious surgical complication, is common after cardiac surgery; however, reports on AKI after noncardiac surgery are limited. We sought to determine the incidence and predictive factors of AKI after gastric surgery for gastric cancer and its effects on the clinical outcomes.

**Methods:**

We conducted a retrospective study of 4718 patients with normal renal function who underwent partial or total gastrectomy for gastric cancer between June 2002 and December 2011. Postoperative AKI was defined by serum creatinine change, as per the Kidney Disease Improving Global Outcomes guideline.

**Results:**

Of the 4718 patients, 679 (14.4%) developed AKI. Length of hospital stay, intensive care unit admission rates, and in-hospital mortality rate (3.5% versus 0.2%) were significantly higher in patients with AKI than in those without. AKI was also associated with requirement of renal replacement therapy. Multivariate analysis revealed that male gender; hypertension; chronic obstructive pulmonary disease; hypoalbuminemia (<4 g/dl); use of diuretics, vasopressors, and contrast agents; and packed red blood cell transfusion were independent predictors for AKI after gastric surgery. Postoperative AKI and vasopressor use entailed a high risk of 3-month mortality after multiple adjustments.

**Conclusions:**

AKI was common after gastric surgery for gastric cancer and associated with adverse outcomes. We identified several factors associated with postoperative AKI; recognition of these predictive factors may help reduce the incidence of AKI after gastric surgery. Furthermore, postoperative AKI in patients with gastric cancer is an important risk factor for short-term mortality.

## Introduction

Although the incidence of gastric cancer has been declining in most advanced nations over the past 2 decades, it remains a major cause of morbidity and mortality worldwide as well as one of the most common malignancies in many east Asian countries [1], [2]. In patients with gastric cancer, perioperative chemotherapy as well as postoperative chemotherapy combined with radiation therapy reduces the recurrence rate and prolongs survival, but surgical resection of the primary tumor and its draining lymph nodes offers the only chance for cure [3], [4]. Various complications (such as pneumonia, wound infection, deep vein thrombosis, and impaired renal function) occur in patients with gastric cancer after gastric surgery [5], [6], [7]. However, the risk factors for postoperative acute kidney injury (AKI) and its effects on the clinical outcomes are not well understood in patients with gastric cancer.

AKI is a serious morbidity occurring during hospitalizations, and it is associated with prolonged hospital stay, high risk of in-hospital mortality and increased hospital costs [8]. In addition, AKI increases the risk of incident and progressive chronic kidney disease and is associated with reduced long-term survival [9]. AKI has been reported to occur in 5–7% of hospitalized patients, but it accounts for up to 20% of admissions in intensive care units (ICUs). Among cases of AKI occurring during hospitalization, approximately 25–40% cases are observed in the operative setting [10], [11], [12]. Although the incidence of postoperative AKI varies with the specific surgical setting, most studies have been performed on postoperative AKI patients undergoing cardiac or vascular surgery. Data on AKI developing in non-cardiovascular surgical settings are limited [13], [14], [15].

We hypothesized that comorbid conditions and perioperative treatments influence the risk for postoperative AKI in gastric cancer patients undergoing gastric surgery. This retrospective study evaluated the incidence and predictive factors of AKI after gastric surgery for gastric cancer, as well as the association between postoperative AKI and clinical outcomes, including mortality and hospital length of stay.

## Materials and Methods

### Study design and patient population

We reviewed the electronic medical records and laboratory results of all adult patients who underwent total or subtotal gastrectomy for gastric cancer in Chonnam National University Hospital between June 1, 2002 and December 31, 2011. Among the 5,160 patients identified, those with insufficient data, emergency operation, chronic kidney disease [preoperative estimated glomerular filtration rate (GFR) <60 mL/(min·1.73 m^2^)] or end-stage renal disease (patients with a history of hemodialysis, peritoneal dialysis, or kidney transplantation) were excluded. We also excluded patients who died within 24 hours of gastric surgery because their mortality was not associated with renal dysfunction and the data were inappropriate for the evaluation of postoperative renal dysfunction. Finally, data from 4,718 patients were analyzed in this study. Cases of death were ascertained by data linkage to the national death certificate database of Statistics Korea and the regional cancer registries. This study was conducted in accordance with the Declaration of Helsinki guidelines. The study protocol was approved by the Institutional Review Board of Chonnam National University Hwasun Hospital in 2013, and informed consent was waived by the Institutional Review Board.

### Data collection and definition

The demographic and perioperative variables assessed were age; sex; heart rate; body mass index; and previous history of hypertension, diabetes mellitus, and chronic obstructive pulmonary disease (COPD). Laboratory data on levels of serum creatinine (SCr), hemoglobin (Hgb), and albumin were extracted from the medical records. The postoperative variables evaluated were use of diuretics, non-steroidal anti-inflammatory drugs (NSAIDs), packed red blood cell (RBC) transfusion, contrast agent, and vasopressors.

AKI was defined according to the Kidney Disease Improving Global Outcomes (KDIGO) clinical practice guidelines. According to these criteria, AKI is present when an abrupt reduction in kidney function results in an absolute increase in SCr level by ≥0.3 mg/dL within 48 hours, an increase of 1.5-fold or more in the baseline SCr level known or presumed to have occurred within prior 7 days, or a reduction in urine output (<0.5 mL/(kg·h) for 6 hours) [16]. We did not consider the urine output criteria because retrospectively collected data have the potential to be inaccurate in this regard. AKI is further classified into 3 stages according to the severity of kidney injury: AKI stage 1, increase in SCr by ≥0.3 mg/dL or 1.5–1.9 times baseline; AKI stage 2, increase in SCr of 2.0–2.9 times baseline; and AKI stage 3, increase in SCr to ≥4.0 mg/dL or ≥3.0 times baseline or initiation of renal replacement therapy. Anemia was defined by the World Health Organization (WHO) diagnostic criteria (Hgb level of <13.0 g/dl and <12.0 g/dl in men and women, respectively) [17]. Preoperative hypoalbuminemia was defined as serum albumin levels of <4.0 g/dL [18]. Smoking was defined as current or ex-smoker. Hypertension was defined by a systolic blood pressure of >140 mmHg, a diastolic blood pressure of >90 mmHg or self-reported hypertension, irrespective of anti-hypertensive medications. Diabetes mellitus was defined by the need for insulin or glucose-lowering medication to control glucose levels on admission, or medical history of diet-controlled diabetes. Intraoperative hypotension was defined by the entry of a systolic blood pressure of <90 mmHg or use of vasopressors (including dopamine, norepinephrine, epinephrine and vasopressin) in the intraoperative anesthesia records.

### Assessment of renal function

SCr levels were analyzed by the Jaffe method, which was calibrated to isotope dilution mass spectrometry. Estimated GFR was calculated with the Chronic Kidney Disease Epidemiology Collaboration (CKD-EPI) equation as follows: mL/(min·1.73 m^2^) = 141×minimum (creatinine/κ, 1)^α^×maximum (creatinine/κ, 1)^−1.209^×0.993^age^×1.018 (if female)×1.159 (if black), where κ is 0.7 for women and 0.9 for men and α is −0.329 for women and −0.411 for men [19].

### Statistical analyses

Continuous variables are expressed as mean ± standard deviation or as medians with interquartile (25^th^ and 75^th^ percentiles) ranges for parametric and nonparametric variables, and categorical variables are presented as the number of patients and percentage. Comparative analysis was performed using Student's *t*-test, analysis of variance, or Kruskal-Wallis test for continuous variables, as appropriate, and Pearson chi-square test for categorical variables. Univariate and multivariate logistic regression analyses were performed to identify the independent predictors of postoperative AKI after gastric surgery. Age, sex and variables with a *P* of <0.1 on univariate analysis were entered into the multivariate logistic regression models. The variables included in the analyses were age; sex; history of hypertension and COPD; smoking; anemia; hypoalbuminemia; use of diuretics, vasopressors, packed RBC transfusion, and contrast agent, intraoperative hypotension; and operation time. An analysis of covariance and multiple logistic regressions adjusted to age and sex was performed to evaluate the association between postoperative AKI and clinical outcomes. We also used logistic regression analyses with backward selection to identify whether postoperative AKI was associated with 3-month mortality after gastric surgery in patients with gastric cancer. All statistical tests were two-tailed, and *P* <0.05 was considered significant. The analyses were performed using the Statistical Package for Social Sciences software, version 17.0 (SPSS, Chicago, Illinois).

## Results

### Risk of Acute Kidney Injury

**Figure 1:**
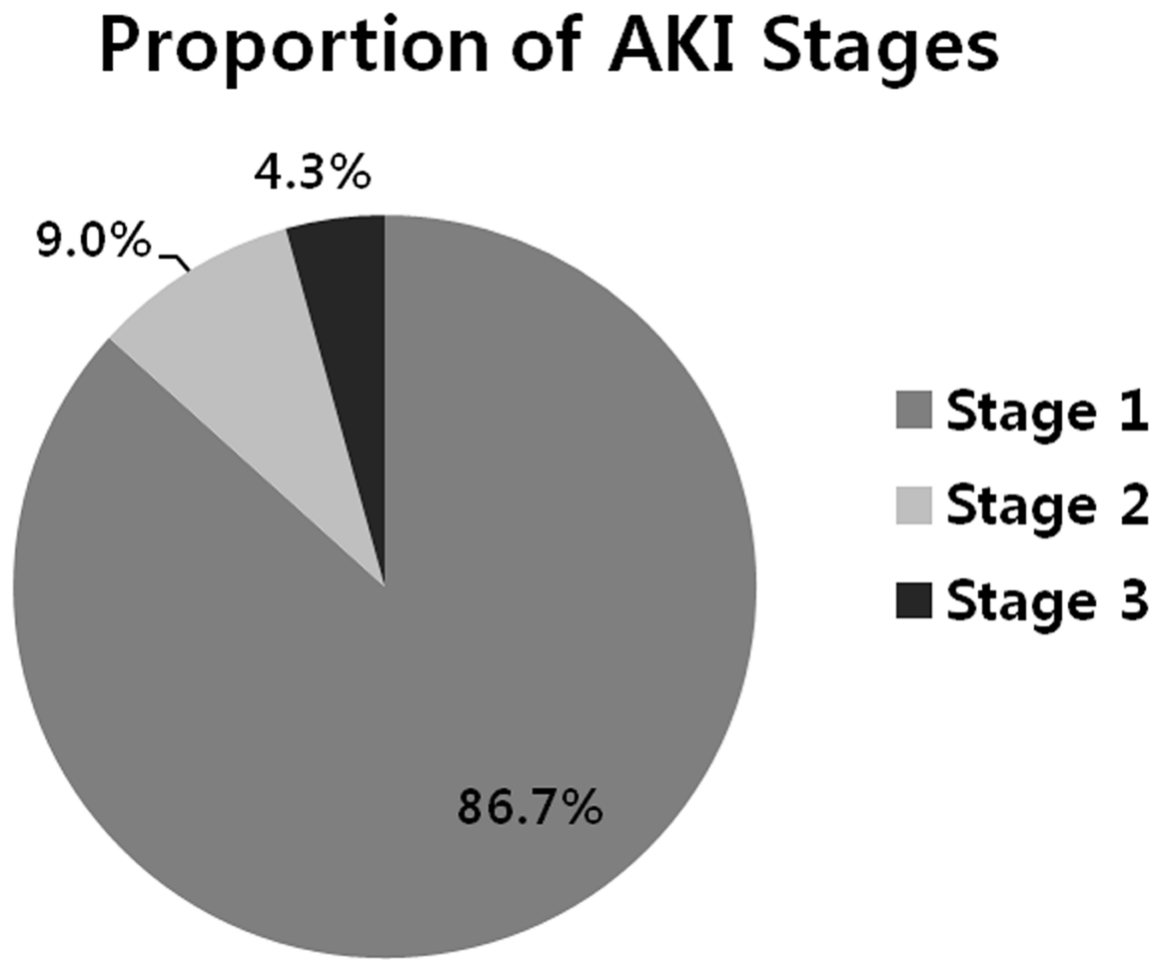
Acute kidney injury (AKI) stages in gastric cancer patients who underwent gastric surgery.

A total 4,718 patients were included in the final analysis. The mean age of the patients was 63.2 years. Of the enrolled patients, 679 patients (14.4%) developed AKI during the postoperative period. Fourteen (2.1%) of the 679 patients developed severe AKI that required renal replacement therapy. According to the KDIGO guideline, 589 of 679 (86.7%) patients were found to have stage 1 AKI; 61 (9.0%) patients, stage 2 AKI; and 29 (4.3%) patients, stage 3 AKI (Figure 1).

The baseline characteristics of patients according to the presence or severity of AKI are listed in Table 1. Patients developing AKI were older and had a higher prevalence of hypertension, COPD, and smoking. They were more likely to have anemia and hypoalbuminemia than patients without AKI. However, there was no difference between the 2 groups in the body mass index and the prevalence of diabetes mellitus. Postoperative use of diuretics, contrast agent, and transfusion of packed RBC were higher in patients with AKI than in those without AKI. Furthermore, the prevalence of anemia and hypoalbuminemia; intraoperative hypotension; use of diuretics, contrast agents, and vasopressin; and transfusion of packed RBCs tended to be higher in patients with higher AKI stage.

**Table 1:** Baseline clinical characteristics.

	All Patients (n = 4718)	No AKI (n = 4039)	AKI (n = 679)^a^	P value h	AKI stage 1^a^ (n = 589)	AKI stage 2 (n = 61)	AKI stage 3 (n = 29)	P value i
Age	63.2±12.1	63.0±12.1	64.5±11.9	0.004	64.1±11.9	66.8±12.1	67.5±10.5	0.084
Male (%)	3171(67.2)	2651(65.6)	520(76.6)	<0.001	453(76.9)	44(72.1)	23(79.3)	0.830
Systolic blood pressure (mmHg)	130±13	129±13	131±14	0.034	130±14	133±13	132±18	0.227
Diastolic blood pressure (mmHg)	83±16	83±17	83±10	0.393	83±10	85±10	84±11	0.435
Heart rate (beats/min)	72±15	72±15	72±16	0.091	73±15	76±16	78±22	0.096
Body mass index (kg/m^2^)	23.3±3.2	23.3±3.2	23.2±3.6	0.520	23.3±3.7	22.8±3.2	22.3±2.5	0.230
Diabetes (%)	684(14.5)	590(14.6)	94(13.8)	0.638	80(13.6)	11(18.0)	3(10.3)	0.903
Hypertension (%) b	1414(30.0)	1177(29.1)	237(34.9)	0.002	206(35.0)	20(32.8)	11(37.9)	0.938
COPD (%)	231(4.5)	162(4.0)	51(7.5)	<0.001	44(7.5)	4(6.6)	3(10.3)	0.747
Smoking (%)	2390(50.7)	2016(49.9)	374(55.1)	0.013	322(54.7)	33(54.1)	19(65.5)	0.381
Hemoglobin (mg/dL)	13.3±2.5	13.4±2.5	13.0±2.2	<0.001	13.1±2.2	12.6±2.4	11.9±2.4	0.005
Anemia (%) c	1304(27.6)	1056(26.2)	248(36.5)	<0.001	207(35.1)	25(41.0)	16(55.2)	0.025
Albumin (g/dL)	4.4(4.1,4.6)	4.4(4.1,4.7)	4.2(3.9,4.5)	<0.001	4.3(3.9,4.5)	4.1(3.6,4.4)	3.9(3.2,4.3)	<0.001
Hypoalbuminemia (%) d	790(16.8)	597(14.8)	193(28.5)	<0.001	154(26.2)	22(36.1)	17(58.6)	<0.001
Baseline creatinine (mg/dL)	0.9±0.2	0.9±0.2	0.9±0.2	0.731	0.9±0.2	0.9±0.2	0.9±0.2	0.242
eGFR (ml/min per 1.73 m^2^) e	98.7±12.4	98.9±12.3	97.4±13.0	<0.001	98.0±12.9	93.7±14.0	93.3±11.1	0.011
Peak creatinine (mg/dL)	1.0±0.4	0.9±0.2	1.5±0.8	<0.001	1.3±0.3	1.9±0.5	4.1±2.2	<0.001
Operation time (hr)	4.07±1.3	4.05±1.3	4.22±1.3	0.001	4.22±1.3	4.22±1.4	4.16±1.0	0.968
Intraoperative hypotension (%) f	990(21.1)	822(20.4)	168(25.1)	0.007	135(23.2)	19(31.1)	14(50.0)	0.001
TNM stage (%)				<0.001				0.001
Stage 1	3059(64.8)	2699(66.8)	360(53.0)		316(53.7)	34(55.7)	10(34.5)	
Stage 2	625(13.2)	516(12.8)	109(16.1)		102(17.3)	4(6.6)	3(10.3)	
Stage 3	576(12.2)	462(11.4)	114(16.8)		93(15.8)	10(16.4)	11(37.9)	
Stage 4	310(6.6)	234(5.8)	76(11.2)		62(10.5)	12(19.7)	2(6.9)	
Diuretics (%)	2033(43.1)	1579(39.1)	454(66.9)	<0.001	383(65.0)	43(70.5)	28(96.6)	0.001
NSAIDs (%)	83(1.8)	68(1.7)	15(2.2)	0.342	13(2.2)	1(1.6)	1(3.4)	0.842
Contrast agent (%)	662(14.0)	482(11.9)	180(26.5)	<0.001	135(22.9)	30(49.2)	15(51.7)	<0.001
p-RBC transfusion (%)	846(17.9)	608(15.1)	238(35.1)	<0.001	183(31.1)	28(45.9)	27(93.1)	<0.001
Postoperative vasopressor use (%) g	190(4.0)	122(3.0)	68(10.0)	<0.001	36(6.1)	16(26.2)	16(55.2)	<0.001

a Defined by Kidney Disease: Improving Global Outcomes guideline.

b Defined by a systolic blood pressure of >140 mmHg, a diastolic blood pressure of >90 mmHg or self-reported hypertension irrespective of anti-hypertensive medications.

c Hemoglobin <13.0 g/dL in men, hemoglobin <12.0 g/dL in women.

d Albumin <4.0 g/dL.

e Estimated GFR, calculated using the Chronic Kidney Disease Epidemiology Collaboration equation.

f Defined by a systolic blood pressure of <90 mmHg or use of vasopressors in the intraoperative anesthesia records.

g Norepinephrine, epinephrine, dopamine, vasopressin, or phenylephrine infusions on postoperative day 1 or 2.

h Compared between and no AKI and AKI.

i Compared among AKI stages.

Abbreviations: AKI, acute kidney injury; COPD, chronic obstructive pulmonary disease; TNM, tumor node metastasis; NSAID, non-steroidal anti-inflammatory drugs; p-RBC, packed red blood cell

Table 2 shows the results of multivariate analysis of predictive factors for AKI after gastric surgery. Multivariate logistic analysis revealed that male gender; hypertension; COPD; hypoalbuminemia; and use of diuretics, vasopressors, packed RBC transfusion, and contrast agents were independently associated with the development of AKI.

**Table 2:** Independent predictors of acute kidney injury after gastric surgery.

	Odds ratio	95% CI	P value
Age (per 10 years)	0.94	0.87–1.02	0.131
Male	1.75	1.37–2.23	<0.001
Hypertension a	1.27	1.04–1.54	0.018
COPD	1.64	1.15–2.35	0.007
Smoking	0.87	0.70–1.08	0.198
Anemia b	0.96	0.77–1.20	0.731
Hypoalbuminemia c	1.40	1.11–1.77	0.005
Diuretics	2.39	1.98–2.88	<0.001
Vasopressors d	1.87	1.28–2.72	0.001
p-RBC Transfusion	1.72	1.38–2.15	<0.001
Contrast agent	1.60	1.29–2.00	<0.001
Intraoperative hypotension e	1.02	0.82–1.28	0.865
Operation time (per hour)	0.97	0.91–1.04	0.452

a Defined by a systolic blood pressure of >140 mmHg, a diastolic blood pressure of >90 mmHg or self-reported hypertension irrespective of anti-hypertensive medications.

b Hemoglobin <13.0 g/dL in men, hemoglobin <2.0 g/dL in women.

c Albumin <4.0 g/dL.

d Norepinephrine, epinephrine, dopamine, vasopressin, or phenylephrine infusions on postoperative day 1 or 2.

e Defined by a systolic blood pressure of <90 mmHg or use of vasopressors in the intraoperative anesthesia records.

Abbreviations: CI, confidence interval; COPD, chronic obstructive pulmonary disease; p-RBC, packed red blood cell.

### Clinical outcomes

**Figure 2:**
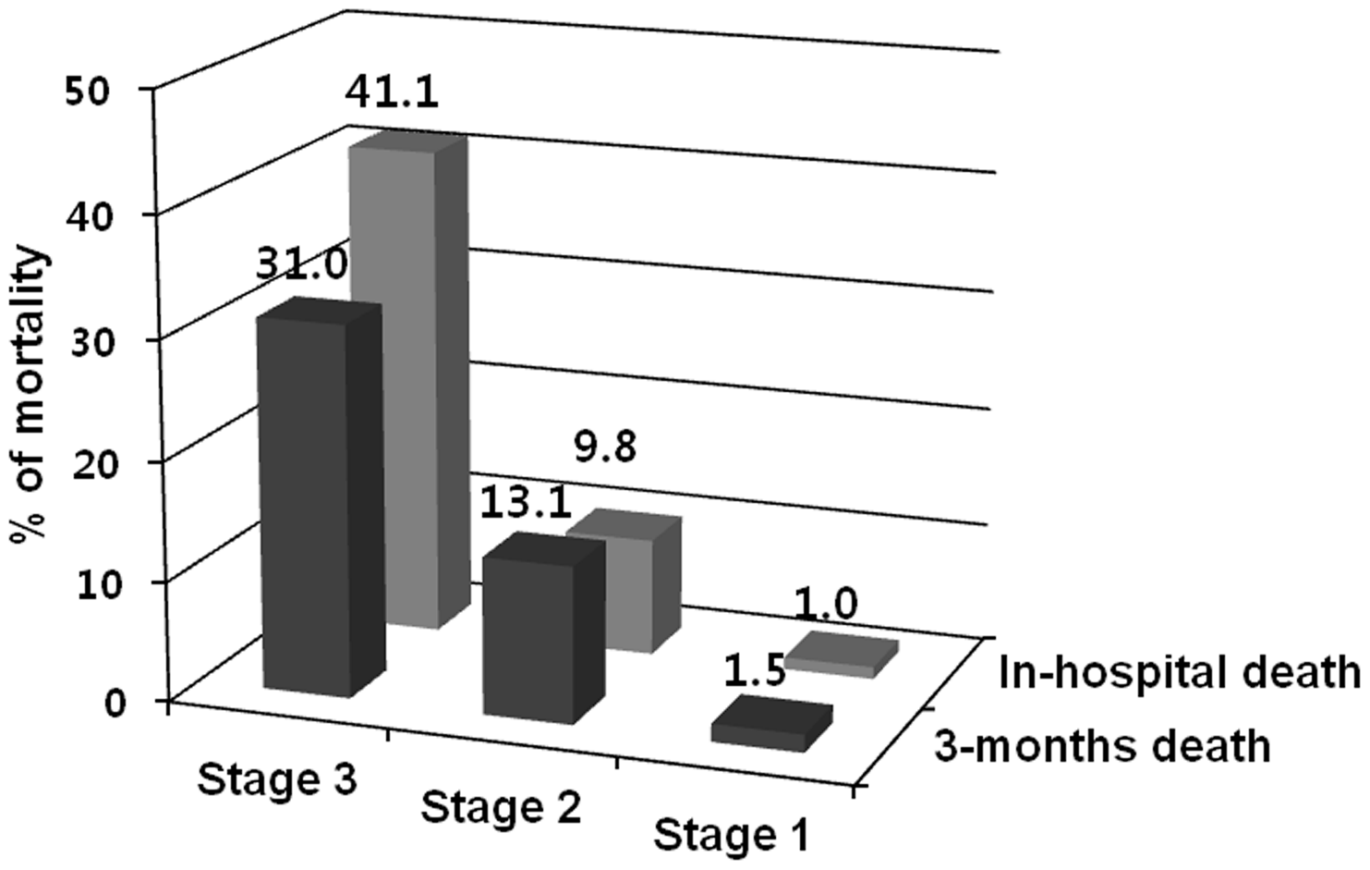
In-hospital and 3-month mortality according to the AKI stages.

As shown in Table 3, the in-hospital and 3-month mortality for patients with AKI were significantly higher than those for patients without AKI (3.5% versus 0.2%, *P*<0.001; 3.8% versus 0.3%, *P*<0.001, respectively). Figure 2 shows the mortality rate according to the AKI stage. The rate of in-hospital and 3-month mortality increased with the advancement in the stage of AKI, in a stepwise manner. In addition, patients with AKI had significantly longer hospital stay and higher prevalence of ICU admission after the operation (mean 18.7 days versus 12.0 days, *P*<0.001; 9.1% versus 1.2%, *P*<0.001, respectively). However, there were no significant differences between the patients with and without AKI in the length of ICU stay.

**Table 3:** Age and sex adjusted clinical outcomes according to AKI after gastric surgery.

	No AKI (n = 4039)	AKI (n = 679)	P value a
In-hospital death (%)	7(0.2)	24(3.5)	<0.001
RRT or CRRT (%)	0(0.0)	14(2.1)	<0.001
Hospital length of stay(days)	12.0±7.1	18.7±20.5	<0.001
ICU care (%)	49(1.2)	62(9.1)	<0.001
ICU length of stay (days)	7.4±17.1	14.0±23.7	0.087
3 months death (%)	11(0.3)	26(3.8)	<0.001

a *P* value by age and sex-adjusted analysis of covariance (ANCOVA) or logistic regression as appropriate.

Abbreviations: AKI, acute kidney injury; RRT, renal replacement therapy; CRRT, continuous renal replacement therapy; ICU, intensive care unit.

We sought to determine whether postoperative AKI was associated with 3-month mortality after gastric surgery in gastric cancer patients by using multivariate logistic regression analysis. The development of postoperative AKI was significantly associated with 3-month mortality after multiple adjustments (OR, 8.75; 95% CI, 3.98–19.27; *P*<0.001), along with age, male gender and use of vasopressors. Low-grade AKI (stage 1), which constituted the majority of postoperative AKI cases in our study, was also an independent predictor for 3-month mortality after gastric cancer surgery (OR, 3.88; 95% CI, 1.47–10.25; *P* = 0.006). Moreover, the relative risks for 3-month mortality increased exponentially as the AKI stage increased (Table 4).

**Table 4:** Independent predictors of 3-month mortality after gastric surgery.

	Odds ratio a	95% CI	P value
Age (per 10 years)	2.40	1.55–3.73	<0.001
Male	9.02	2.20–36.95	0.002
Vasopressors	5.96	2.56–13.91	<0.001
AKI	8.75	3.98–19.27	<0.001
AKI stage 1	3.88	1.47–10.25	0.006
AKI stage 2	25.47	8.50–76.31	<0.001
AKI stage 3	58.80	16.70–207	<0.001

Abbreviations: AKI, acute kidney injury.

a Conditional logistic regression adjusted by age, gender, hypertension, COPD, smoking, diabetes mellitus, anemia, hypoalbuminemia, the use of diuretics, vasopressors, and RBC transfusion, intraoperative hypotension, gastric cancer stage, and AKI stage.

## Discussion

In this study, we determined the incidence, predictive factors, and outcomes for AKI, defined according to the recently published and validated KDIGO guidelines, occurring after gastric surgery in patients with gastric cancer. We found that 14.4% of these patients developed postoperative AKI and that 2.1% of these patients required renal replacement therapy. Male gender, hypertension, COPD, hypoalbuminemia, and use of diuretics, vasopressors, packed RBC transfusion and contrast agents were identified as independent predictors for postoperative AKI. Furthermore, in-hospital and 3-month mortality increased with the severity of AKI, and patients developing AKI had a higher risk of 3-month mortality after gastric surgery.

Postoperative AKI remains a leading cause of morbidity, mortality, prolonged hospital stay, and increased hospital cost [10], [20]. The reported incidence of postoperative AKI varies from 0.8% to 30%, depending on the definition of AKI, the preoperative renal function of the patients, and the various surgery types considered in the respective studies [15], [21], [22]. Although data available on the incidence of AKI after noncardiac surgery are lesser than those available on AKI after cardiovascular surgery, the incidence of postoperative AKI in our study was comparable to that noted in previous studies [13], [14]. One study revealed that an incidence of 8.5% for AKI, defined as either a 50% increase in SCr level or requirement of dialysis, developing after gastric bypass surgery for morbid obesity [13]. Another study reported an incidence of 30% for AKI in patients undergoing liver transplantation and indicated that the frequency of severe AKI requiring dialysis can be as high as 17% [14]. However, a recent study [15] showed a relatively low incidence of postoperative AKI (less than 1%) in patients with normal kidney function undergoing noncardiac surgery. In that study, AKI was defined by an absolute level of estimated GFR (<50 mL/min) within the first 7 postoperative days, which is a highly restrictive criterion for the detection of AKI. Consequently, the underestimation of the incidence of postoperative AKI in the abovementioned study compared to ours may be attributed to the difference in the definitions of AKI in both the studies. Unfortunately, as mentioned earlier, there is no uniformity in the definition of AKI adopted in the various studies. Recently, KDIGO proposed new criteria wherein AKI is defined by alterations in the SCr level within 7 days. Therefore, the KDIGO criteria can detect AKI in patients with slow increase in creatinine and may also be a better predictor of mortality and represent a standardized, simple method of categorizing AKI [23]. We thought that the new KDIGO criteria were useful for the precise diagnosis of postoperative AKI.

Our findings demonstrated that hypertension, COPD and hypoalbuminemia were independent preoperative predictors for AKI after gastric surgery. Kheterpal *et al* [15]. have reported preoperative AKI risk factors such as old age, emergency surgery, liver disease, high body mass index, high-risk surgery, peripheral vascular disease, and COPD, but they did not evaluate the preoperative status of laboratory parameters, including albumin and Hgb, under non-cardiovascular surgical settings. Indeed, hypoalbuminemia has been shown to be an important risk factor for AKI in various clinical conditions [14], [24], [25], [26]. Another study on patients who underwent off-pump coronary bypass surgery revealed that a low preoperative serum albumin level (<4.0 g/dL) is independently associated with postoperative AKI, as observed in our study [18]. Although the underlying mechanisms by which hypoalbuminemia causes the development of postoperative AKI are not completely understood, albumin appears to not only improve renal perfusion through prolonged potent renal vasodilatation but also inhibits the apoptosis of renal tubular cells through its capacity to scavenge reactive oxygen species [27], [28]. Consequently, albumin plays a crucial role in the maintenance of proximal tubular integrity and function. Therefore, low level of serum albumin may contribute to an increased risk of postoperative AKI in patients undergoing gastric surgery.

Several intraoperative management variables were found to be independent predictors of AKI. Use of vasopressors and diuretics is associated with increased postoperative AKI, as reported in a previous study [15]. Although it is unclear whether use of diuretics is a cause or a consequence of postoperative AKI, indiscriminate use of diuretics to treat postoperative low urine output should be avoided, especially in patients with hypovolemic status. Interestingly, transfusion of packed RBCs may also have deleterious effects on renal outcomes, because the shortened lifespan of the preserved RBC results in circulating free iron-mediated nephrotoxicity with hemolysis and free Hgb, as well as induction of oxidative stress [29], [30]. Those are important mechanisms of AKI. We also found that packed RBC transfusion was associated with postoperative AKI in the multivariate analysis, as reported in previous studies [31], [32]. This finding suggests that unnecessary blood transfusions should be avoided in patients undergoing gastric surgery. However, anemia and intraoperative hypotension were not associated with AKI in this study, which is in contrast to the results previously reported [31], [33]. A potential reason for this discrepancy might be attributed to the relatively higher level of mean Hgb (13.3 mg/dL) in our study than in the previous one [31]. In other words, severe anemia, particularly in combination with intraoperative hypotension might contribute to renal injury, thereby highlighting a potential synergic effect. Indeed, arterial hypoxemia or hypotension alone would not lead to renal injury, possibly because of the compensating effect of increased renal blood flow or decreased renal oxygen consumption [34]

AKI without supporting renal replacement therapy is associated with increased mortality in critically ill patients and in patients who have undergone surgery [10], [11], [15]. Our findings also showed that in-hospital and 3-month mortality, and prevalence of ICU care in patients with AKI were higher than those in patients without AKI, and the values of these factors increased in a stepwise manner with the severity of the injury defined as per the KDIGO criteria. It appears that more severe AKI is associated with a higher risk for morbidity and mortality in patients undergoing gastric surgery. Although the 3-month mortality recorded in this study was lower than reported previously for noncardiac surgeries of different types [15], it should be noted that in both studies, the patients who developed AKI had significantly high mortality rates. Furthermore, our results revealed that patients with postoperative AKI had an 8.75-fold higher risk of 3-month mortality compared to those without AKI. Postoperative AKI has never been tested as a predictor of mortality after gastric surgery in gastric cancer patients, as in our study; thus, our study provides valuable, independent information. In addition, the duration of hospitalization, which has been recognized as a surrogate indicator of costs, was significantly greater in postoperative AKI patients compared with those without AKI after gastric surgery. Thus, AKI can be viewed as a distinct therapeutic target, the prevention and treatment of which may improve the clinical outcomes of surgery.

The strengths of this study are the relatively large number of patients, use of recent and validated AKI definitions for the assessment of clinical outcomes, and inclusion of various perioperative predictors. Our study also has several limitations. First, we used only the change in the SCr level to determine the occurrence of AKI because exact data regarding urine output were not available for this retrospective study. This may have resulted in underestimation of the incidence of AKI. Second, despite our best efforts to adjust our results for most confounders, the results of this study may have been affected by residual confounding.

## Conclusion

AKI was common after gastric surgery for gastric cancer, and several factors were associated with the development of postoperative AKI. Postoperative AKI in patients with gastric cancer is a potent predictive factor for adverse clinical outcomes after gastric surgery. Therefore, identifying predictive factors and preventing the development of postoperative AKI are essential for improving patient survival.
